# Branched chain fatty acid synthesis drives tissue-specific innate immune response and  infection dynamics of  *Staphylococcus aureus*

**DOI:** 10.1371/journal.ppat.1009930

**Published:** 2021-09-08

**Authors:** Xi Chen, Wei Ping Teoh, Madison R. Stock, Zachary J. Resko, Francis Alonzo

**Affiliations:** Department of Microbiology and Immunology, Loyola University Chicago–Stritch School of Medicine, Maywood, Illinois, United States of America; University of Tubingen, GERMANY

## Abstract

Fatty acid-derived acyl chains of phospholipids and lipoproteins are central to bacterial membrane fluidity and lipoprotein function. Though it can incorporate exogenous unsaturated fatty acids (UFA), *Staphylococcus aureus* synthesizes branched chain fatty acids (BCFA), not UFA, to modulate or increase membrane fluidity. However, both endogenous BCFA and exogenous UFA can be attached to bacterial lipoproteins. Furthermore, *S*. *aureus* membrane lipid content varies based upon the amount of exogenous lipid in the environment. Thus far, the relevance of acyl chain diversity within the *S*. *aureus* cell envelope is limited to the observation that attachment of UFA to lipoproteins enhances cytokine secretion by cell lines in a TLR2-dependent manner. Here, we leveraged a BCFA auxotroph of *S*. *aureus* and determined that driving UFA incorporation disrupted infection dynamics and increased cytokine production in the liver during systemic infection of mice. In contrast, infection of TLR2-deficient mice restored inflammatory cytokines and bacterial burden to wildtype levels, linking the shift in acyl chain composition toward UFA to detrimental immune activation *in vivo*. In *in vitro* studies, bacterial lipoproteins isolated from UFA-supplemented cultures were resistant to lipase-mediated ester hydrolysis and exhibited heightened TLR2-dependent innate cell activation, whereas lipoproteins with BCFA esters were completely inactivated after lipase treatment. These results suggest that *de novo* synthesis of BCFA reduces lipoprotein-mediated TLR2 activation and improves lipase-mediated hydrolysis making it an important determinant of innate immunity. Overall, this study highlights the potential relevance of cell envelope acyl chain repertoire in infection dynamics of bacterial pathogens.

## Author summary

Fatty acids present in the bacterial cell envelope are diverse and represent key components of membrane phospholipids and membrane-anchored lipoproteins, the latter of which are potent inducers of antibacterial innate immunity. While *S*. *aureus* exclusively synthesizes saturated straight and branched chain fatty acids, it can also acquire host unsaturated fatty acids from the host environment. In this study, we examined how changes in *S*. *aureus* fatty acid composition alter innate immune responses and infection dynamics. Our results indicate that a shift in membrane fatty acid repertoire away from branched chain fatty acids and toward unsaturated fatty acids results in tissue-specific disruption of infection dynamics that correlate with lipoprotein-mediated induction of innate immunity. *De novo* branched chain fatty acid synthesis by *S*. *aureus* shifts the balance away from host unsaturated fatty acid-induced inflammation and stabilizes bacterial burden during infection. Thus, acyl chain composition represents a facet of the bacterial cell envelope that calibrates the immune response and bacterial infection outcome.

## Introduction

*Staphylococcus aureus* synthesizes or scavenges essential nutrients and evades immune responses to promote persistence during infection [1–5]. One such nutrient is fatty acids [6–11]. Fatty acids are essential to the structure and function of bacterial phospholipids and lipoproteins and serve to maintain membrane homeostasis [7,12–14]. *S*. *aureus* synthesizes saturated straight chain fatty acids (SFA) as well as branched chain fatty acids (BCFA) via the branched-chain 2-oxoacid dehydrogenase (BCODH) complex and fatty acid synthase II (FASII) [10,11,15–17]. BCFA confer membrane fluidity and are essential for *S*. *aureus* viability [10,16]. In contrast, host membrane fatty acids comprise SFA and unsaturated fatty acids (UFA). UFA are the primary agent of membrane fluidity in mammalian cells [18]. Unlike mammalian cells, *S*. *aureus* does not synthesize UFA, but it can incorporate them from exogenous sources [19,20].

*De novo* fatty acid synthesis via FASII constitutes up to 95% of the energy used for membrane phospholipid synthesis in *S*. *aureus* [21]. Thus, the ability to scavenge exogenous fatty acids (eFA) may allow the cell to redirect energy use in response to shifts in the environmental nutrient supply. eFA incorporation by *S*. *aureus* requires the fatty acid kinase, FakA, and fatty acid-binding proteins FakB1 and FakB2, where FakB1 preferentially binds SFA and FakB2 binds UFA [20,22,23]. FakA subsequently phosphorylates the eFA for FASII elongation or direct assimilation into membrane phospholipids [24]. Incorporation of eFA from the host environment can support bacterial survival in the presence of FASII inhibitors through bypass of *de novo* fatty acid synthesis [12,19,25,26]. In addition, studies with human serum and murine thigh infection imply a degree of UFA incorporation by *S*. *aureus* even in the absence of FASII inhibitors [8,9]. Beyond restoring viability during FASII inhibition, the functional consequences of flexibility in membrane fatty acid composition on the virulence of *S*. *aureus* is unknown. However, cultured *S*. *aureus* can incorporate free UFA onto bacterial lipoproteins to promote Toll-like receptor 2 (TLR2) signaling *in vitro* and aminoacylation with long chain fatty acids is known to reduce TLR2 signaling compared to short chain fatty acids [27–29]. While eFA uptake is a beneficial trait that promotes energy conservation in response to the nutritional environment, the incidental induction of inflammation because of UFA uptake could also represent an advantageous host defense mechanism that occurs secondary to nutrient uptake.

Most host fatty acids are stored as ester-linked appendages within triglycerides, phospholipids, and cholesterol esters [30]. These lipids are assembled and transported to a range of tissue sites via the bloodstream in lipoprotein particles. The most abundant circulating lipoprotein particle in humans is low-density lipoprotein (LDL) [31–35]. Upon reaching host tissues, lipids must be processed by host lipases (ester hydrolases) that release fatty acids and cholesterol for energy, incorporation into membranes, or use as second messengers [36–39]. *S*. *aureus* also secretes at least two lipases to hydrolyze fatty acid esters from host sources [19,40–42]. In addition, the *S*. *aureus* glycerol ester hydrolase, Geh, acts on bacterial lipid-anchored proteins to hydrolyze fatty acid esters and mask TLR2-mediated signaling [43]. Recent reports suggest that Geh targets host LDL *in vitro* to release eFA for maintenance of membrane integrity during steady-state, FASII inhibition, or BCFA auxotrophy [5,8,19].

Here, we leveraged a BCFA auxotroph of *S*. *aureus* to determine how acyl chain composition of the bacterial membrane contributes to inflammation during infection. We found that driving host UFA incorporation during murine infection leads to increased bacterial burden in the liver, but not other organs. *In vitro* analyses determined that UFA incorporation via FakB2 rendered *S*. *aureus* lipoproteins poor substrates for lipase-mediated inactivation and led to increased TLR2-dependent immune cell activation. This manifested *in vivo* as heightened TLR2-dependent inflammatory cytokine secretion (irrespective of bacterial CFU) and dramatically disrupted infection burden in the livers of mice infected with a BCFA auxotroph, but not WT *S*. *aureus*. Infection of TLR2-deficient mice restored cytokines and bacterial burden to levels observed during infection of WT mice with WT *S*. *aureus*. These results imply that UFA incorporation and TLR2 activation leads to pathologic inflammation that dictates bacterial infection dynamics. In contrast, *de novo* BCFA synthesis by *S*. *aureus* shifts the balance away from host UFA-induced inflammatory responses and stabilizes bacterial burden during infection. Thus, acyl chain composition represents a facet of the bacterial cell envelope that calibrates the immunological response and establishes bacterial infection outcome.

## Results

### Growth in GTO and LDL enhances immune cell activation by *S*. *aureus* culture supernatant

**Fig 1:**
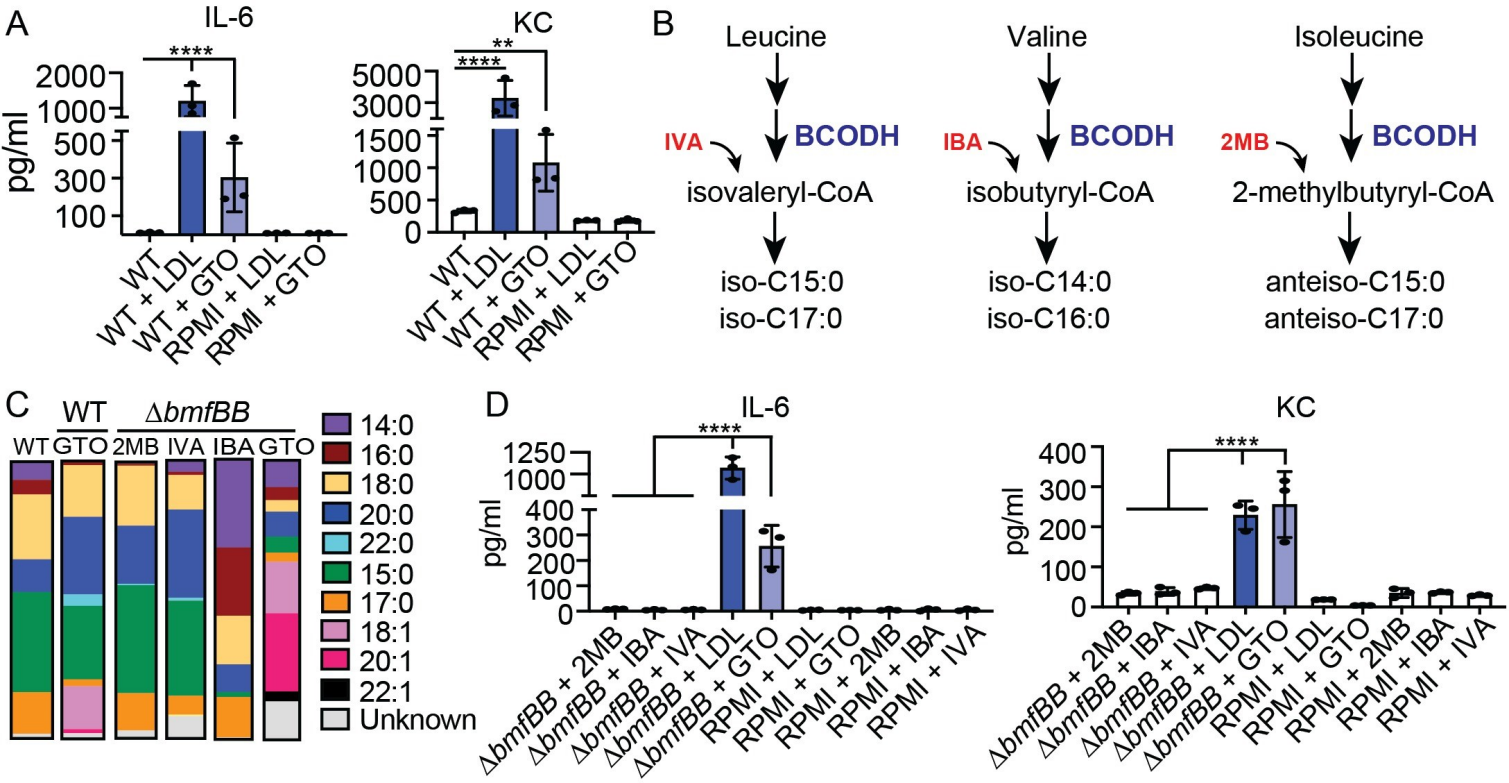
Culture in LDL and GTO enhances immune cell activation by *S*. *aureus*. (A) IL-6 and KC (pg/mL) production by BMMs after addition of cell-free supernatant from WT *S*. *aureus* grown in RPMI medium, RPMI medium supplemented with human LDL (0.34 μg/μL) or GTO (50 μM), and media only controls. (B) BCFA biosynthesis from branched amino acid precursors. BCODH, branched chain 2-oxoacid dehydrogenase; IVA, isovaleric acid; IBA, isobutyric acid; 2MB, 2-methylbutyric acid. (C) GC-FAME analysis of WT, Δ*bmfBB*, or Δ*bmfBB* grown in the presence of 2MB (9 mM), IVA (9 mM), IBA (10 mM), and GTO (50 μM). Each color represents the proportion of the indicated fatty acid from the total population. Data is representative from one of 2–3 independent GC-FAME analyses. (D) IL-6 and KC (pg/mL) production by BMMs after addition of cell-free supernatant from Δ*bmfBB* mutant *S*. *aureus* grown in RPMI medium, RPMI medium supplemented with 2MB (9 mM), IVA (9 mM), IBA (10 mM), human LDL (0.34 μg/μL) and GTO (50 μM), or media only controls. Data from cytokine assays display mean +/- SD from one of at least three independent experiments conducted in triplicate. Statistical significance was determined by one-way ANOVA with Tukey’s post hoc test. ** *P* < 0.01; **** *P* < 0.0001.

eFA uptake by *S*. *aureus* provides a means to divert energy associated with FA synthesis to other biological process. The incorporation of free UFA onto bacterial lipoproteins by *S*. *aureus* also leads to enhanced TLR2 signaling, which may represent an important host defense strategy [28]. Host fatty acids are commonly stored in lipoprotein particles such as LDL or in lipid droplets where they are esterified to glycerol (triglycerides, TAG) [31–36,44,45]. Thus, we tested if growth of *S*. *aureus* in media supplemented with human LDL or glyceryl trioleate (GTO), a common mammalian triglyceride, might also enhance immunostimulatory capacity of *S*. *aureus* culture supernatant [46,47]. Cultures of WT *S*. *aureus* were grown in RPMI medium supplemented with human LDL or GTO and cell free supernatant derived from these cultures was applied onto murine bone marrow derived macrophages (BMM). All strains grew identically under these conditions (~1–2 x 10^9^ CFU/mL) regardless of lipid supplementation [5]. We observed that cell free supernatant isolated from *S*. *aureus* grown in the presence of LDL and GTO led to significant increases in the production of interleukin-6 (IL-6) and keratinocyte chemoattractant (KC) compared to supernatant from cultures of WT bacteria grown in RPMI medium without LDL and GTO or media alone supplemented with LDL or GTO (Fig 1A).

*S*. *aureus* synthesizes BCFA *de novo*, precluding the ability to evaluate the effects of UFA incorporation in the absence of endogenous BCFA synthesis [9,20]. We recently generated a Δ*bmfBB* mutant of *S*. *aureus*, which lacks the gene encoding the E2 subunit of the branched-chain 2-oxoacid dehydrogenase (BCODH) complex and is deficient for the synthesis of BCFA (Fig 1B) [5,17]. A Δ*bmfBB* mutant is unable to grow in broth unless supplemented with branched chain carboxylic acids [isobutyric acid (IBA), isovaleric acid (IVA), and 2-methylbutyric acid (2MB)] (Fig 1B), BCFA, or UFA [5]. Thus, the fatty acid composition of a Δ*bmfBB* mutant can be altered by varying the types of fatty acids and fatty acid precursors present in the growth medium [11,17]. Indeed, fatty acid composition analysis of a Δ*bmfBB* mutant grown in the presence of GTO followed by gas chromatography-fatty acid methyl-ester (GC-FAME) analysis demonstrated a shift toward a predominance of unsaturated and saturated straight chain fatty acids (50% UFA and 40% SFA) with residual BCFA (<10%) compared to supplementation with IBA, IVA, or 2MB (Fig 1C) [17,48]. This contrasted with WT cells supplemented with GTO, which harbored ~15% UFA, but maintained a considerable proportion of BCFA (~30%). Supernatant from cultures of a Δ*bmfBB* mutant grown in RPMI medium supplemented with LDL and GTO led to significant increases in the production of IL-6 and KC from BMM compared to supplementation with 2MB, IBA, and IVA (Fig 1D). Together, these data indicate LDL and GTO supplementation of WT or Δ*bmfBB* mutant *S*. *aureus* cultures is immunostimulatory. Furthermore, GTO supplementation of a Δ*bmfBB* mutant shifts the fatty composition of this strain toward UFA, an effect that coincides with enhanced immune cell activation by *S*. *aureus* culture supernatant compared to cultures supplemented with BCFA precursors.

### UFA from host lipid stores increases lipoprotein TLR2 signaling

**Fig 2:**
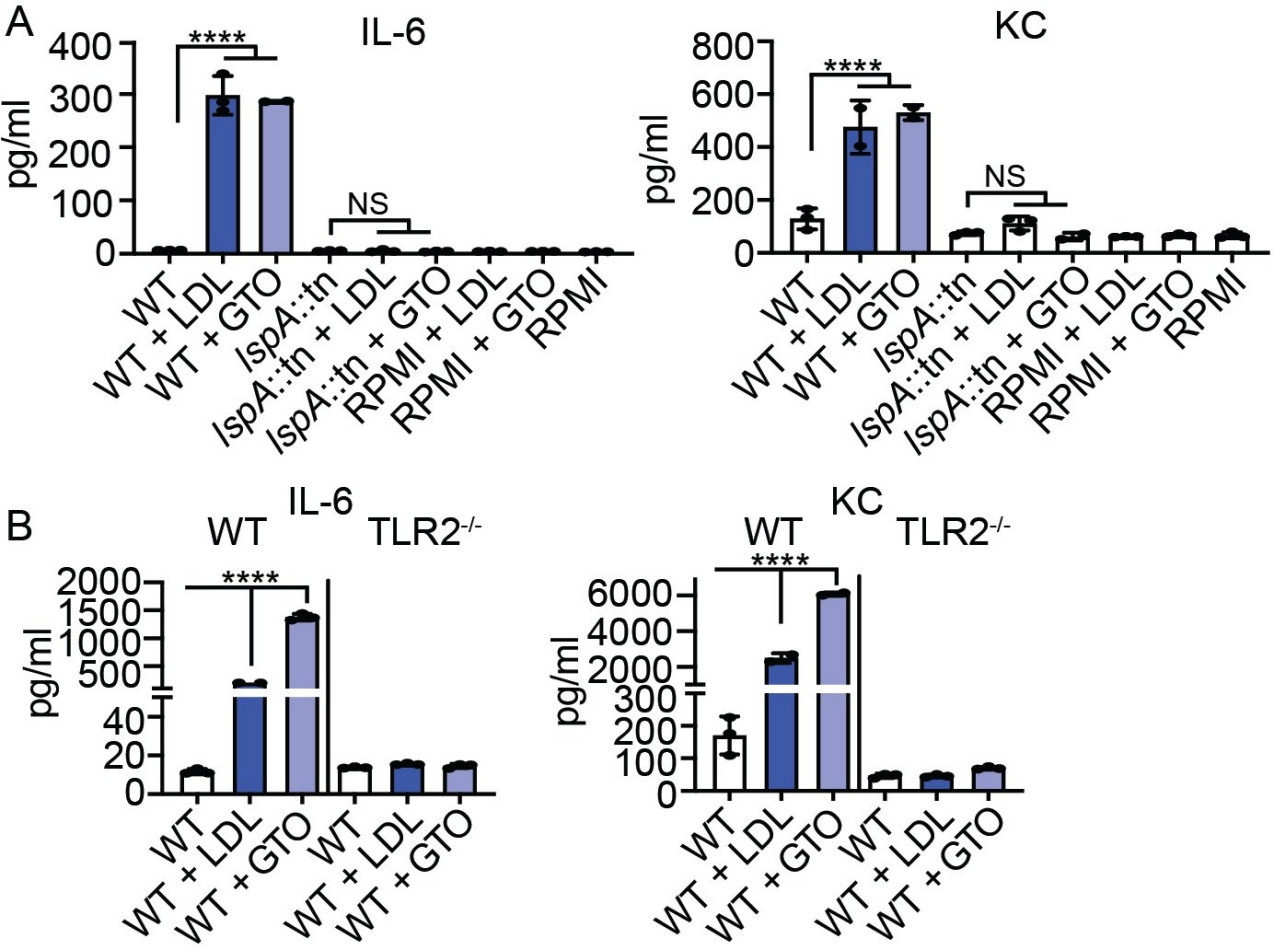
UFA from host lipid stores increases lipoprotein TLR2 signaling. (A) IL-6 and KC (pg/mL) production by BMMs after addition of cell-free supernatant from WT *S*. *aureus* or an *lspA*::*tn* mutant grown in RPMI medium, RPMI medium supplemented with human LDL (0.34 μg/μL) or GTO (50 μM), and media only controls. (B) IL-6 and KC (pg/mL) production by WT and TLR2^-/-^ BMMs after addition of cell-free supernatant from WT *S*. *aureus* or an *lspA*::*tn* mutant grown in RPMI medium, RPMI medium supplemented with human LDL (0.34 μg/μL) or GTO (50 μM), and media only controls. Graphs display mean +/- SD from one of at least three independent experiments conducted in triplicate. Statistical significance was determined by one-way ANOVA with Tukey’s post hoc test. **** *P* < 0.0001. NS, Not Significant.

Nguyen et al. previously demonstrated that attachment of free UFA onto lipoproteins or synthetic lipopeptides promotes TLR2 activation in mammalian cell lines [28]. We reasoned that macrophage activation caused by WT and Δ*bmfBB* mutant supernatant upon supplementation with LDL and GTO was due to the transfer of UFA onto lipoproteins. To confirm that incorporation of UFA from lipid stores enhanced TLR2 activation via *S*. *aureus* lipoprotein acyl chains, we applied supernatants from human LDL and GTO-supplemented cultures of a lipoprotein maturation-deficient mutant, *lspA*::*tn*, to BMM and quantified inflammatory cytokines [49,50]. Supernatant from the *lspA*::*tn* mutant was unable to induce cytokine production by BMM regardless of lipid supplementation (Fig 2A). We then tested if activation of BMM occurred directly through TLR2 by applying supernatant from LDL and GTO-supplemented cultures of WT *S*. *aureus* to *TLR2*^*-/-*^ BMM. *TLR2*^-/-^ cells had significantly reduced production of IL-6 and KC even when bacteria were grown in the presence of LDL and GTO (Fig 2B). These data indicate UFA from host lipid stores enhance lipoprotein signaling through TLR2.

### Roles of lipases and the UFA binding protein in eFA-mediated immune cell activation

**Fig 3:**
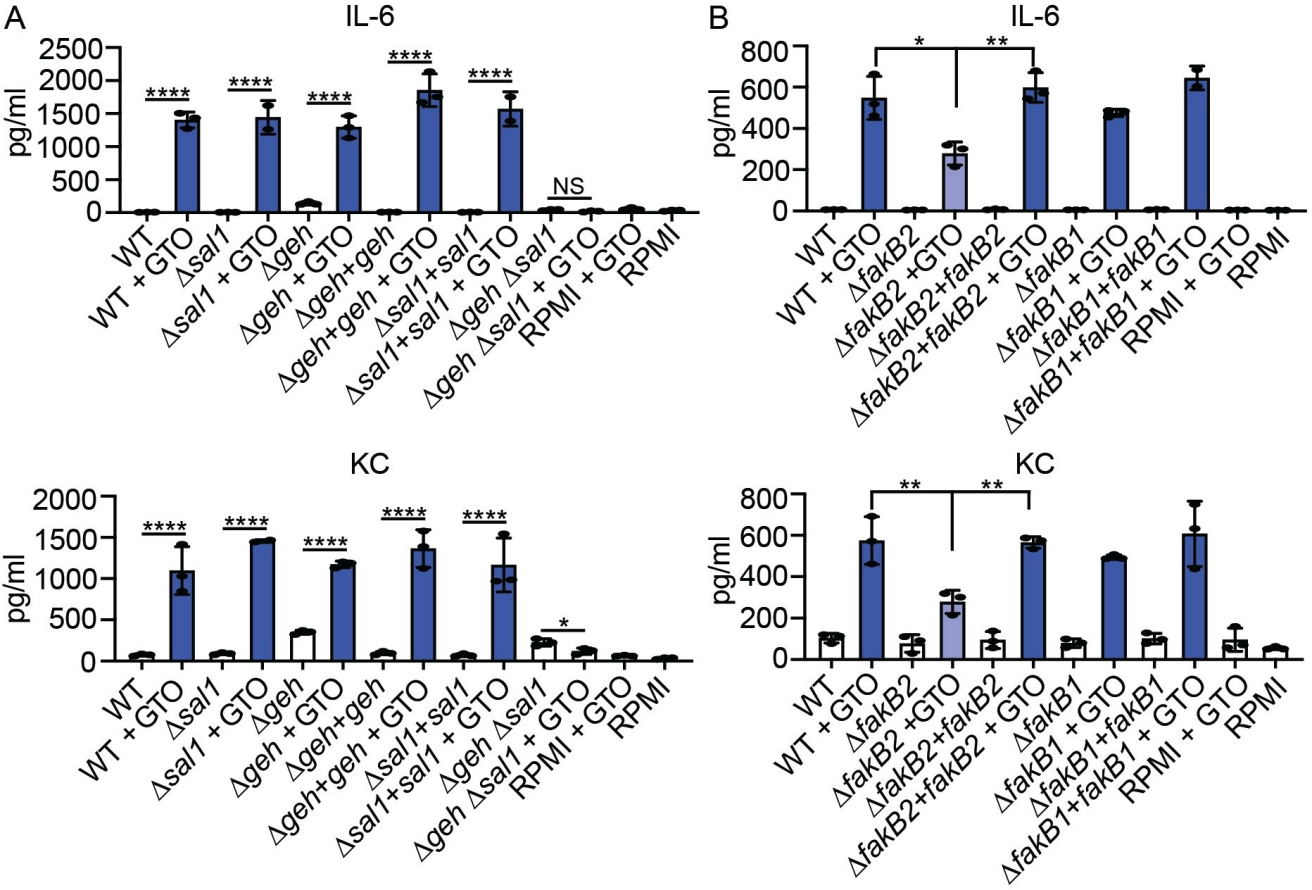
Lipases and FakB2 mediate eFA-based immune cell activation. (A-B) IL-6 and KC (pg/mL) production by BMMs after addition of cell-free supernatant from the indicated *S*. *aureus* strains grown in RPMI medium or RPMI medium supplemented with GTO (50 μM), and media only controls. Graphs display mean +/- SD from one of at least three independent experiments conducted in triplicate. Statistical significance was determined by one-way ANOVA with Tukey’s post hoc test. ** *P* < 0.01; **** *P* < 0.0001.

Fatty acids are most often found esterified to glycerol and cholesterol in lipid stores, such as circulating lipoprotein particles and lipid droplets [31–36,44,45]. *S*. *aureus* secretes two abundant ester hydrolases, Geh (Sal2) and Sal1, of which Geh was shown to facilitate the acquisition of fatty acids from LDL as a nutrient source [5,19,40]. To determine the role of lipases in releasing and incorporating esterified immunostimulatory UFA, we supplemented cultures of WT, Δ*geh*, Δ*sal1*, Δ*geh* Δ*sal1*, Δ*geh*+*geh*, and Δ*sal1*+*sal1 S*. *aureus* with GTO and applied supernatant to macrophages followed by monitoring cytokine production (Fig 3A). In keeping with our prior studies, supernatant from a Δ*geh* mutant grown in RPMI without GTO supplementation elicited higher cytokine production due to the absence of Geh-mediated hydrolysis of bacterial lipoproteins (6.5 pg/mL versus 140.7 pg/mL of IL-6 and 74.5 pg/mL versus 349.1 pg/mL of KC for WT and Δ*geh* mutant supernatant respectively). However, upon supplementation with GTO, we observed elevated IL-6 and KC production after treatment of BMM with supernatant derived from all strains except for the Δ*geh* Δ*sal1* double mutant (Fig 3A). Together, these data indicate that, during *in vitro* growth, both Geh and Sal facilitate UFA release and promote macrophage activation.

*S*. *aureus* eFA acquisition is mediated by FakB1 and FakB2, where FakB2 binds preferentially to UFA [20,22,23]. We surmised that UFA incorporation by FakB2 contributed to the enhancement in BMM activation. Indeed, the addition of supernatant derived from a Δ*fakB2* mutant cultured in the presence of GTO partially reduced levels of IL-6 and KC compared to WT, Δ*fakB1*, Δ*fakB1*+*fakB1*, and Δ*fakB2*+*fakB2* strains (Fig 3B). Altogether, these results indicate that FakB2-mediated binding of UFA contributes to enhanced BMM activation *in vitro*.

### *S*. *aureus* lipoprotein is the source of UFA-mediated immune cell activation

**Fig 4:**
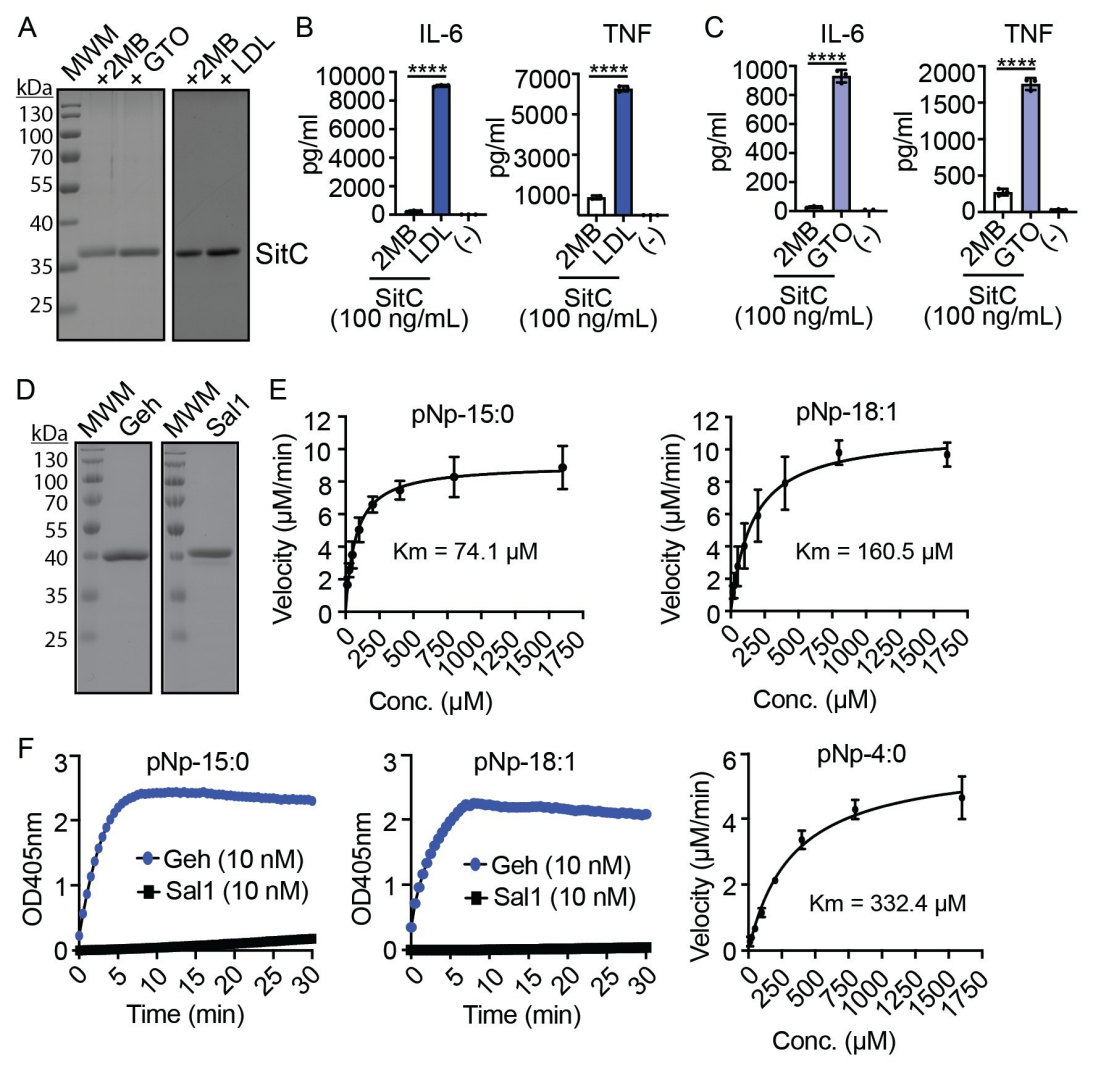
Lipoprotein-mediated immune cell activation and lipase activity on pNp fatty acid esters. (A) GelCode Blue-stained 12% SDS-PAGE gels of 5 μg SitC-6xHis purified from a Δ*bmfBB* mutant grown in RPMI supplemented with 2MB (9 mM), eyLDL (0.1%), or GTO (50 μM). (B-C) IL-6 and TNF production by BMM after addition of SitC (100 ng/mL) purified from the growth media indicated in (A). (-), media alone. (D) GelCode Blue-stained 12% SDS-PAGE gels of 5 μg purified recombinant Geh-6xHis and Sal1-6xHis. (E) Michaelis-Menten curves depicting the rate of pNp-15:0 or pNp-18:1 hydrolysis at varying concentrations of substrate. Kinetic assays were performed in triplicate and the average reaction rates were determined from at least 6 independent assays. Km was calculated from the combined dataset. (F) pNp-15:0, and pNp-hydrolysis in the presence of 10 nM Geh or Sal1 and Michaelis-Menten curve depicting the rate of pNp-4:0 hydrolysis at varying concentrations of substrate. Data from cytokine assays display mean +/- SD from one of at least three independent experiments conducted in triplicate. Statistical significance was determined by one-way ANOVA with Tukey’s post hoc test. **** *P* < 0.0001.

Thus far, our data indicate that bacterial culture supernatant supplemented with LDL or GTO enhances immune activation, presumably because of the release of bacterial lipoproteins containing UFA. To confirm lipoprotein as the source of immune cell stimulation, we purified 6xHis-tagged SitC, one of the most abundant lipoproteins in *S*. *aureus*, from a Δ*bmfBB* mutant grown in medium supplemented with either egg yolk LDL, GTO, or 2MB (Fig 4A). The addition of purified SitC from egg yolk LDL or GTO-supplemented cultures to BMM resulted in a substantial increase in IL-6 and TNF production by BMM compared to the addition of SitC from 2MB-supplemented cultures (Fig 4B and 4C). KC levels after treatment with recombinant SitC could not be reliably determined on account of measurements that fell outside the working range of the assay. These data support the notion that enhanced immune cell activation derived from GTO and LDL supplementation is ascribed to *S*. *aureus* lipoproteins and is consistent with studies using free UFA [28].

### Assessment of Geh and Sal1 acyl chain specificity

Our data and that of others suggest UFA attachment onto lipoproteins promotes TLR2 signaling [28]. Yet, recent evidence suggests Geh (Sal2) can inactivate *S*. *aureus* lipoproteins through hydrolysis of ester-linked fatty acids to reduce TLR2-mediated innate immunity [43]. Data in Fig 4A indicate that, when grown in the presence of GTO, WT and Δ*geh* mutant *S*. *aureus* supernatant elicits identical amounts of IL-6 and KC from macrophages, suggesting that Geh may not inactivate bacterial lipoproteins under these conditions. We reasoned this discrepancy could be due to the specificity of Geh for saturated versus unsaturated fatty acid esters on bacterial lipoprotein. Therefore, we determined the kinetics of Geh and Sal1 (Fig 4D) ester hydrolysis in the presence of para-nitrophenol (pNp) conjugated substrates containing either anteiso-C15:0 BCFA or C18:1 UFA. Geh had a lower *K*_m_ for pNp-anteiso-C15:0 (74.1 μM) compared to pNp-C18:1 (160.5 μM) (Fig 4E). In contrast, we were unable to detect substantial Sal1-mediated hydrolysis of either pNp substrate within 30 minutes (Fig 4F). Indeed, it is known that Sal1 preferentially cleaves short chain fatty acids [40,51,52]. We confirmed Sal1 activity using pNp-butyrate (C4:0) as a substrate (*K*_m_ 332.4μM) (Fig 4F). Together, our data suggest Geh exhibits a moderately higher affinity for anteiso-C15:0 compared to C18:1 whereas Sal1 does not readily hydrolyze these pNp substrates [40,52].

### Geh does not inactivate *S*. *aureus* lipoproteins derived from UFA cultures

**Fig 5:**
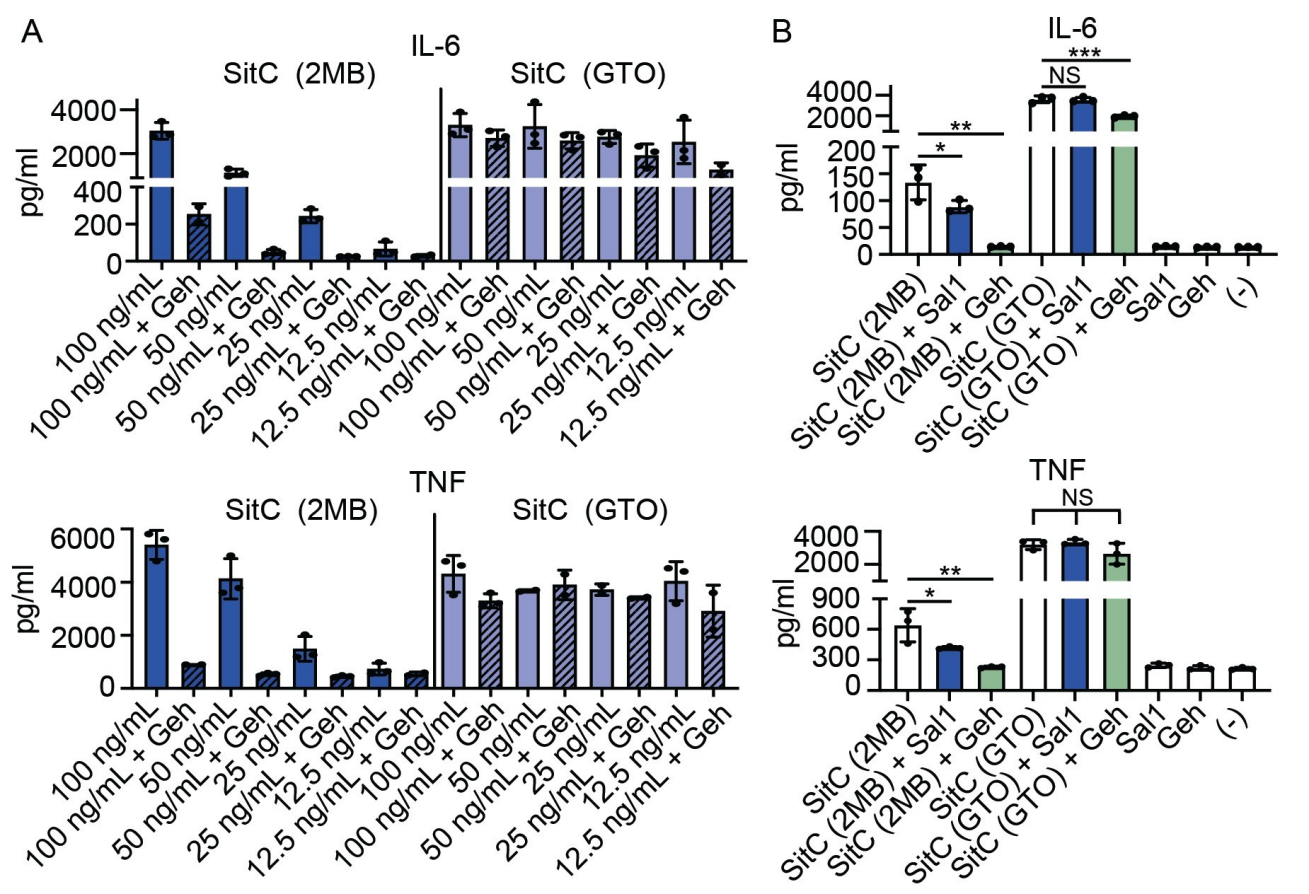
Geh does not inactivate *S*. *aureus* lipoproteins with UFA moieties. (A) IL-6 and TNF production by BMM after addition of SitC (varying concentrations (ng/mL)) purified from a Δ*bmfBB* mutant grown in RPMI supplemented with 2MB (9 mM) or GTO (50 μM) that was either untreated or treated with Geh (20 μM SitC: 1 μM Geh). (B) IL-6 and TNF production by BMM after addition of SitC (100 ng/mL) purified from a Δ*bmfBB* mutant grown in RPMI supplemented with 2MB (9 mM) or GTO (50 μM) that was either untreated or treated with Sal1 or Geh (20 μM SitC: 1 μM Sal1 or Geh). Data from cytokine assays display mean +/- SD from one of at least three independent experiments conducted in triplicate. Statistical significance for (B) was determined by one-way ANOVA with Tukey’s post hoc test. * *P* < 0.05; ** *P* < 0.01; *** *P* < 0.001.

The aforementioned pNp substrates contained single acyl chains and thus do not fully resemble a native *S*. *aureus* lipoprotein substrate, which is di- or triacylated [53–55]. Thus, we wondered if Geh acyl chain hydrolysis would be further impacted in the context of an *S*. *aureus* lipoprotein. To test this possibility, we again purified SitC from a Δ*bmfBB* mutant cultured in 2MB (BCFA) and GTO (UFA) followed by incubation with Geh. We observed that Geh treatment significantly reduced the induction of IL-6 and TNF by SitC from 2MB-supplemented cultures, but not SitC from GTO-supplemented cultures (Fig 5A). In addition, titration of Geh-treated SitC derived from GTO-supplemented cultures revealed maximal BMM activation at a range of concentrations, whereas Geh-treated SitC derived from 2MB-supplemented cultures was rendered inactive (Fig 5A). Of note, the purified SitC used for experiments in Fig 5A elicited an equivalent cytokine response regardless of 2MB or GTO supplementation at the highest concentration applied (100 ng/mL), however SitC derived from GTO-supplemented cultures exhibited far greater potency at lower concentrations. Despite circumstantial evidence of Sal1 targeting host lipid stores (Fig 3A), we found that Sal1 treatment of SitC derived from 2MB-supplemented cultures only partially reduced the production IL-6 and TNF compared to treatment with Geh, which completely eliminated cytokine production (Fig 5B). Sal1 or Geh treatment of SitC derived from GTO-supplemented cultures had minimal effect on IL-6 or TNF levels, though the modest reduction in IL-6 for Geh-treated SitC achieved statistical significance (Fig 5B). Overall, these findings imply that bacterial lipoproteins containing BCFA are not only less potent inducers of TLR2 but also have a greater propensity for inactivation by Geh.

### *S*. *aureus* virulence and use of UFA

**Fig 6:**
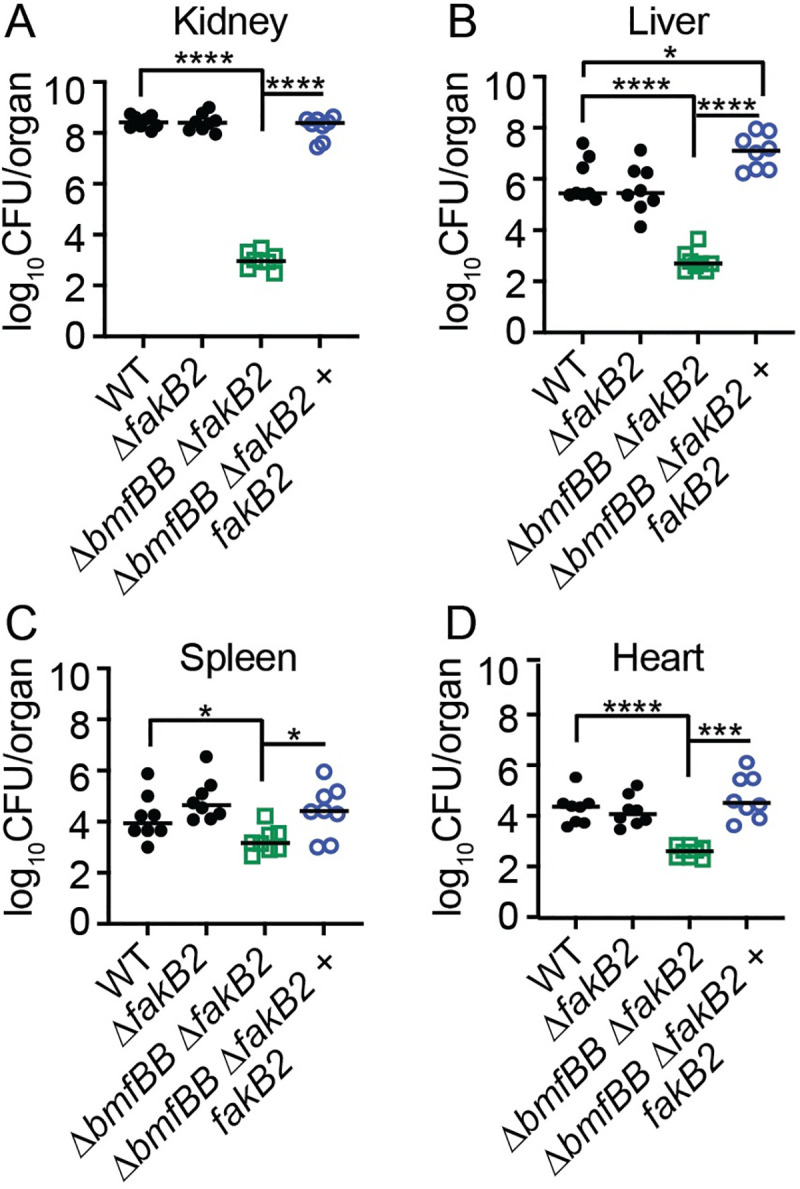
*S*. *aureus* bypass of BCFA auxotrophy. (A-D) Bacterial burden (log_10_ CFU per organ) recovered from kidney (A), liver (B), spleen (C), and heart (D) 72 hours post-infection with WT, Δ*fakB2*, Δ*bmfBB* Δ*fakB2*, and Δ*bmfBB* Δ*fakB2* + *fakB2* (*n* = 8 per group). Graphs represent combined data from two independent experiments. Statistical significance was determined Kruskal-Wallis test with Dunn’s posttest. * *P* < 0.05; *** *P* < 0.001, **** *P* < 0.0001.

*S*. *aureus* incorporates UFA from the host environment via the fatty acid binding protein FakB2 [20,22]. To evaluate the effects of UFA incorporation on *S*. *aureus* pathogenesis, we infected five- to six-week-old C57BL/6 mice, via the bloodstream, with WT, Δ*fakB2*, Δ*fakB2* Δ*bmfBB*, and Δ*fakB2* Δ*bmfBB* + *fakB2* strains. After 72 hours, mice infected with a Δ*fakB2* mutant had bacterial burdens in the kidneys, liver, spleen, and heart that were comparable to those in mice infected with WT *S*. *aureus* (Fig 6). However, mice infected with a Δ*fakB2* Δ*bmfBB* mutant had a significantly reduced burden in all organs (Fig 6). The magnitude of attenuation was most pronounced in kidney and liver with 10,000 and 1,000-fold fewer colony forming units (CFU), respectively (Fig 6A and 6B) compared to ~10-fold in spleen and heart (Fig 6C and 6D). Infection with a Δ*fakB2* Δ*bmfBB* + *fakB2* complement strain fully restored *S*. *aureus* virulence in kidneys, spleen, and heart (Fig 6A, 6C and 6D). The results in the kidney fully replicate outcomes reported in our prior studies [5]. Surprisingly, we noted a 50-fold increase in CFU recovered from infected livers, a central site of fatty acid metabolism (Fig 6B) [56–58]. Altogether, these results suggest that during systemic infection a BCFA auxotroph (Δ*fakB2* Δ*bmfBB* + *fakB2*) is hypervirulent in the liver, but not other organs.

### UFAs drive innate immune activation to alter infection dynamics

**Fig 7:**
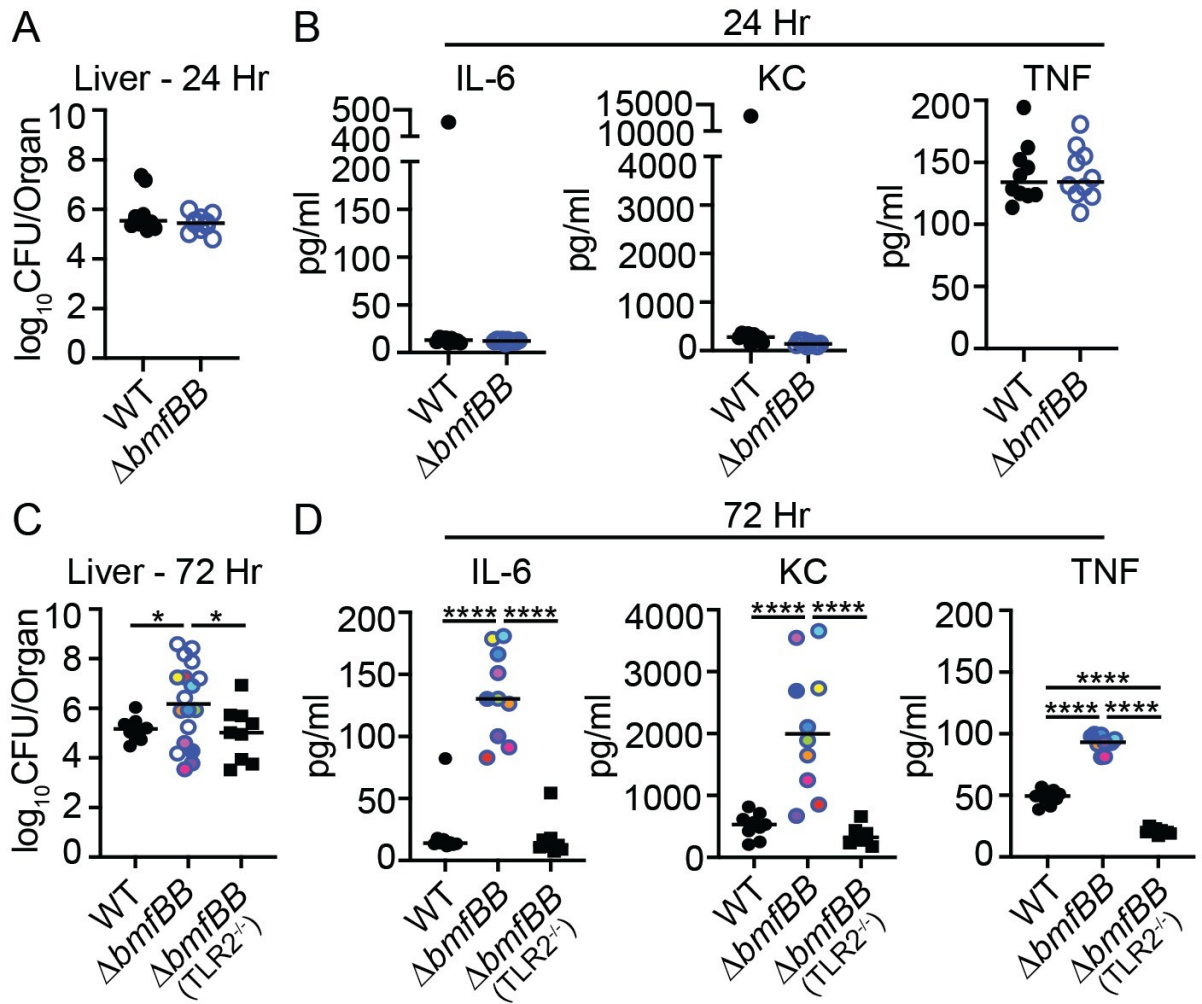
UFAs drive innate immune activation to alter infection dynamics. (A) Bacterial burden (log_10_ CFU per organ) recovered from liver 24 hours post-infection with WT or Δ*bmfBB* mutant *S*. *aureus* (*n* = 10 per group). (B) IL-6, KC, and TNF levels (pg/mL) in liver 24 hours post-infection with the strains indicated in (A). (C) Bacterial burden (log_10_ CFU per organ) recovered from liver 72 hours post-infection of WT mice with WT *S*. *aureus* (*n =* 10), or WT (*n =* 18) and TLR2^-/-^ (*n* = 9) mice with Δ*bmfBB* mutant *S*. *aureus*. (D) IL-6, KC, and TNF levels (pg/mL) in liver 72 hours post-infection. Ten Δ*bmfBB* liver homogenates were sampled from one of the replicates from (C) and matching colors indicate the cytokine levels from an individual mouse. Graphs represent the combined data of at least two independent experiments. Statistical significance for (C) was determined using a Kolmogorov-Smirnov test and statistical significance for (D) was determined by one-way ANOVA with Tukey’s post hoc test. * *P* < 0.05; **** *P* < 0.0001.

The hypervirulence observed in the livers of mice infected with the Δ*fakB2* Δ*bmfBB* + *fakB2* strain (Fig 6) coupled with enhanced cytokine secretion by BMM treated with supernatant of a BCFA auxotroph grown in the presence of LDL and TAG (Fig 1) led us to wonder if driving UFA incorporation with a BCFA auxotroph *in vivo* leads to TLR-mediated disturbances in innate immunity that disrupt host infection dynamics. We infected WT or TLR2^*-/-*^ mice with a Δ*bmfBB* mutant and compared CFU and tissue cytokines to WT mice infected with WT *S*. *aureus*. At 24 hours, identical CFU were recovered from mice infected with WT and Δ*bmfBB* mutant *S*. *aureus* (Fig 7A). In addition, there were no notable differences in the average levels of IL-6 (~12 pg/mL), KC (~200 pg/mL) and TNF (~135 pg/mL) in liver homogenates (Fig 7B). In contrast, a Δ*bmfBB* mutant had a dramatically altered liver infection pattern at 72 hours post-infection (Fig 7C). Two-thirds (12/18) of the infected animals had 10-1000-fold increased CFU, whereas six mice had equivalent to 10-fold fewer CFU, representing a nearly six-log spread in the data (Fig 7C). We sampled 10 animals representing low and high CFU counts to measure cytokine levels and found that liver homogenates from all animals had significantly increased levels of IL-6, KC, and TNF regardless of CFU burden (Fig 7D). In contrast, TLR2^*-/-*^ mice infected with a Δ*bmfBB* mutant had CFU and cytokine levels that closely resembled infection of WT mice with WT *S*. *aureus* after 72 hours (Fig 7C and 7D). These data suggest that UFA uptake by *S*. *aureus in vivo* establishes an inflammatory environment that disrupts infection dynamics in the liver.

## Discussion

*S*. *aureus* can assimilate environmental UFA into its membrane lipids and lipoproteins in place of endogenously derived fatty acids [9,12,25]. *S*. *aureus* lipoproteins with UFA or synthetic triacylated lipopeptides containing unsaturated acyl chains cause enhanced TLR2 signaling [28,59]. However, the relevance of maintaining an optimal BCFA to UFA ratio as it relates to *S*. *aureus* pathobiology is not fully understood. Here, we showed that the lipase, Geh, mediates the hydrolysis of host lipids to release UFA, which the bacterium can incorporate in a Fak-dependent manner, leading to immune activation that perturbs infection dynamics. This immunostimulatory phenotype is derived from bacterial lipoproteins that become poor substrates for Geh ester hydrolysis. One possibility is that *S*. *aureus* maintains a preference for BCFA over UFA to mitigate these untoward immunostimulatory effects, thereby establishing an immune environment that reduces inflammation and drives consistent infection dynamics. Thus, the acyl chain repertoire of the membrane is a key facet of *S*. *aureus* immunomodulation and virulence.

The enhanced proinflammatory response observed for a BCFA auxotroph grown in the presence of GTO compared to BCFA precursors suggests that shifting the BCFA to UFA ratio is crucial to defining immunological responses *in vitro* and potentially infection dynamics *in vivo*. However, the extent and context of UFA incorporation by wildtype *S*. *aureus* is less well defined [9]. Indeed, *de novo* fatty acid synthesis consumes a large amount of energy and as such, *S*. *aureus* may adapt to acquire eFAs *in vivo* when nutrients are limiting or when demand for lipid synthesis is high [11,19]. In support of this argument, a BCFA auxotroph (Δ*bmfBB*) survives on host UFA in a FakB2-dependent manner in certain tissue sites, suggesting host UFA can substitute for BCFA to promote bacterial survival (Fig 6 and [5]). This is consistent with several prior studies that showed host fatty acids allow *S*. *aureus* to bypass FASII inhibitors to infect mice [12,19,25,60,61]. Recent studies using a murine thigh infection model also demonstrated some assimilation of host UFA by WT *S*. *aureus* via FakB2 [9]. While assimilation of UFA *in vivo* was observed in this infection model, a significant amount of BCFA remained, suggesting that WT *S*. *aureus* could favor BCFA over UFA in the membrane during infection [9]. Our data support this notion as we observed limited UFA-mediated immune activation during infection of the liver with WT *S*. *aureus*, whereas supplementation of *in vitro* cultures of WT *S*. *aureus* with excess GTO is highly immunostimulatory (Figs 1 and 7). However, it should be noted that the thigh and liver represent two markedly different infection sites, where the impact and extent of eFA incorporation could vary making direct comparisons challenging. Intriguingly, the liver was the only infected organ where we observed lipid assimilation-dependent changes in inflammation and virulence. The liver and adipose tissue are the two primary sites of lipid metabolism [56,57]. We suspect environments that are rich in fatty acid biogenesis are likely to have the most significant impact on *S*. *aureus* fatty acid repertoire. Another infectious site that contains excess fatty acids is the skin. An in-depth interrogation of immunological responses in the skin on account of UFA uptake awaits investigation in light of this work and prior observations [5]. Nevertheless, it is intriguing that infection of the liver dramatically shifted infection dynamics, with most animals exhibiting greater infection burdens (Fig 7). Though one might intuit an increase in inflammation brought about by infection with a Δ*bmfBB* mutant would be beneficial to the host, as it could lead to infection clearance, our data indicate this is not the case in the liver. We suspect this finding signifies a critical balance exists in the magnitude and duration of an innate immune response that could determine beneficial and detrimental infection outcomes. It is possible that the hyperinflammatory response induced by the Δ*bmfBB* mutant leads to a dysregulated immune phenotype that tips the balance away from the host in favor of the bacterium. Future studies are needed to test this possibility.

Our data suggest the possibility that *S*. *aureus* does not require UFA for survival *in vivo* (Fig 6). This observation implies that *de novo* BCFA synthesis is predominant during infection and potentially limits bacterial acquisition of UFA. This notion is supported by previous studies indicating that culture of WT *S*. *aureus* in the presence of oleic acid (18:1) does not appreciably change BCFA content, as approximately 50% of membrane is still composed of BCFA [11,62]. Furthermore, the sn-2 position of *S*. *aureus* phospholipid is exclusively occupied by BCFA in most conditions, implying an established preference for BCFA [9,16,20]. Thus, together with our *in vitro* data, it appears that BCFA synthesis is advantageous to *S*. *aureus* on account of: (1) competition with host UFA during membrane phospholipid biogenesis, and (2) increased attachment of BCFA to bacterial lipoproteins, rendering them less immunostimulatory on account of Geh hydrolysis of BCFA esters [43].

The observation that *S*. *aureus* lipoproteins containing UFA enhance immune activation seems to contradict prior studies suggesting that Geh inactivates bacterial lipoproteins via hydrolysis of ester-linked acyl chains [43]. However, Geh ester hydrolysis assays suggest that enhanced stimulation of TLR2 by *S*. *aureus* lipoproteins harboring UFA is attributed to the reduced affinity for unsaturated acyl chains (Figs 4 and 5). Indeed, the *K*_m_ of Geh for BCFA versus UFA-containing pNp esters indicated that Geh had a moderately higher affinity for pNp-anteiso 15:0 compared to pNp-18:1 (Fig 4). In addition, lipoproteins isolated from a BCFA auxotroph supplemented with GTO were largely insensitive to Geh-mediated inactivation (Fig 5). We suspect the difference between pNp esters and lipoproteins is due to the context in which the acyl chains are presented to Geh. In prior studies, structural variations at sn-2 acyl chains of triacylglycerides were found to alter the stereoselectivity of purified microbial lipases [63]. *S*. *aureus* lipoproteins contain two ester-linked acyl chains derived from a phosphatidylglycerol precursor, while pNp-conjugated ester substrates only have one acyl chain. Thus, it is possible that the sn-2 acyl chain of lipoproteins contributes to the low substrate preference of Geh for lipoproteins with UFA attachments.

Geh is able to liberate UFA from host lipid stores *in vivo* and *in vitro* despite limited activity on bacterial lipoproteins containing these same substrates [5,19]. We postulate that the difference in activity is due to the structural variation between bacterial lipoproteins and host lipid stores. Lipid droplets and lipoprotein particles of the host are enriched in phospholipids, cholesterol esters, and triacylglycerols [31–36,44,45], the latter of which is a common substrate for Geh [8,40,64]. Our *in vitro* analyses do not ascertain substrate preferences of Geh for these alternative host lipid stores and thus represents a limitation of the study. We are currently devising methods to test preferences for these more complex substrates.

Thus far, our data support the prevailing model that *S*. *aureus* liberates and acquires host UFA from lipid stores via lipases and the fatty acid binding protein FakB2 [19,20,22]. However, our *in vitro* data suggest FakB2 is not the only fatty acid binding protein responsible for incorporating exogenous UFA (Fig 3). We attribute this discrepancy to the *in vitro* experimental growth conditions, which include incubation for 8 hours in medium supplemented with 50 μM GTO. It is possible these conditions may allow for compensation by FakB1, which typically recognizes SFA [20]. We attempted to test this hypothesis using a Δ*fakB1* Δ*fakB2* double mutant, however we observed enhanced immune cell activation that was independent of lipid supplementation and closely resembled a Δ*fakA* mutant, which has known pleiotropic effects. Therefore, we excluded the Δ*fakB1* Δ*fakB2* double mutant from these studies [62,65–67]. In a similar vein, we found that both Geh and Sal1 contributed to the generation of an eFA pool from GTO (Fig 3). This observation contrasts with studies of Delekta et al., who showed Geh, but not Sal1, liberates fatty acids from LDL in the presence of FASII antibiotics [19]. It is possible that the lack of Sal1 activity observed in previous studies is because Sal1-mediated FA acquisition is not sufficient for FASII bypass given its comparatively lower activity on long chain fatty acid esters [40,51,52]. We suspect the role for Sal1 in GTO hydrolysis may be limited to the *in vitro* studies described in this work; however, it could manifest in a lipid-enriched environment of the host such as the liver and adipose tissue where substrates are potentially in excess. We are currently testing this possibility.

In sum, UFA scavenging by *S*. *aureus* has the potential to reduce its energy and metabolite expenditures, which could prove valuable for infection. However, shifting the balance of BCFA to UFA in the cell envelope can have significant impacts on innate immunity and infection dynamics at sites of lipid metabolism. As such, *S*. *aureus* appears to favor BCFA biosynthesis for persistence during infection. Overall, this study expands our understanding of how *S*. *aureus* interacts with the host lipid environment during infection.

## Methods

### Ethics statement

All experiments were performed following an IACUC-approved protocol (IACUC #2020025) that adheres to the guidelines of the Office of Laboratory Animal Welfare, USDA and PHS policy, and the ethical standards of the Institutional Biosafety Committee and the Institutional Animal Care and Use Committee (IACUC) at Loyola University Chicago Health Sciences Division. The institution is approved by Public Health Service (PHS; A3117-01 through 02/28/2022), is fully accredited by the AAALAC International (000180, certification dated 11/17/2016), and is registered/licensed by USDA (33-R-0024 through 08/24/2023).

### Bacterial strains and growth conditions

The strains used in this study are described in Table A in S1 Text. All *S*. *aureus* strains were grown in Tryptic Soy Broth (TSB) (BD Biosciences) or Roswell Park Memorial Institute 1640 (RPMI) medium (Corning) at 37°C with shaking at 200 rpm unless otherwise noted. For experiments that required supplementation with lipids or lipid precursors, *S*. *aureus* strains were first grown overnight in RPMI and then subcultured into RPMI supplemented with 0.1% egg yolk LDL, 0.34 μg/μl human LDL (Kalen Biomedical), 50 μM glycerol trioleate (GTO, Sigma-Aldrich), or branched chain carboxylic acids that are precursors to BCFA including 9 mM isovaleric acid (IVA, Sigma-Aldrich), 10 mM isobutyric acid (IBA, Sigma-Aldrich), and 9 mM 2-methylbutyric acid (2MB, Alfa Aesar). For total lipid extraction studies, overnight bacterial cultures from strains grown in RPMI or RPMI supplemented with 50 μM GTO, 9 mM IVA, 10 mM IBA, or 9 mM 2MB were inoculated 1:100 into the same medium followed by growth for eight hours at 37°C with shaking at 200 rpm. *Escherichia coli lysY/I*^*q*^ was used for Geh and Sal1 purification. *E*. *coli* DH5α and DC10B were used for propagation of pIMAY, pJC1111, pJC1112, pQE60, and pOS1 plasmids. All *E*. *coli* strains were cultivated in Lysogeny Broth, Miller formulation (BD Biosciences) at 37°C with shaking at 200 rpm. Antibiotics were used when necessary. For *E*. *coli* strains: 100 μg/mL ampicillin (Gold Biotechnology). For *S*. *aureus* strains: 10 μg/mL chloramphenicol (Amresco) and 3.5 μg/mL erythromycin (Amresco).

### Construction of Δ*fakB1* + *fakB1* and Δ*fakB2* + *fakB2* complement strains

The Δ*fakB1 + fakB1* and Δ*fakB2 + fakB2* complement strains were generated using the pJC1111 plasmid [68]. Using primers listed in Table B in S1 Text, the *fakB1* and *fakB2* genes were amplified from WT *S*. *aureus* genomic DNA, while the constitutive *P*_HELP_ promoter was amplified from the pIMAY plasmid [69]. The resulting amplicons were used in a splicing by overlap extension (SOE) PCR to obtain *P*_HELP_-*fakB1* and *P*_HELP_-*fakB2*, which were cloned into pJC1111 at the PstI and SalI restriction endonuclease cut sites. The recombinant plasmids were propagated in *E*. *coli* DH5α and transformed into SaPI-1 integrase-expressing *S*. *aureus* (RN9011) to allow single-copy integration in the chromosome at the SaPI-1 site [68,70]. Bacteriophage Φ11 was used for packaging the integrated complementation plasmids from RN9011, followed by transduction into the Δ*fakB1* and Δ*fakB2* strains as previously described [70]. Δ*fakB1 + fakB1* and Δ*fakB2 + fakB2* transductants were selected on 0.15 mM cadmium chloride and confirmed by PCR.

### Generation of Geh-6xHis and Sal1-6xHis expressing *E*. *coli lysY/I*^*q*^

A Geh-6xHis expression plasmid was previously generated [43]. A Sal1-6xHis expression plasmid was generated using the expression plasmid pQE60 (Qiagen). The gene sequence corresponding to mature Sal1 (amino acids K282-A680) was amplified by PCR from WT *S*. *aureus* genomic DNA using primer pair pQE60-Sal1-NcoI and pQE60-Sal1-BglII (Table B in S1 Text) followed by digestion with NcoI and BglII endonucleases and subsequent ligation into the pQE60 plasmid. The pQE60-Sal1-6xHis plasmid was propagated in *E*. *coli* DH5α, then transformed into T7 expressing *E*. *coli lysY/I*^*q*^ for induction and purification.

### Purification of Geh-6xHis, Sal1-6xHis

Geh-6xHis and Sal1-6xHis were purified as previously described [43].

### Endotoxin removal of Geh-6xHis, Sal1-6xHis

Endotoxin removal from purified recombinant Geh-6xHis and Sal1-6xHis was conducted as previously described with some modifications [71]. Briefly, Triton X-114 was added to purified lipase to a final concentration of 2% (vol/vol), followed by incubation at 4°C on a rotisserie. After two hours, the sample was transferred to a 37°C water bath for 10 minutes followed by centrifugation at 20,000 × g for 20 min at 37°C. The protein-containing upper layer was separated and collected. This process was repeated twice. Bio-Beads SM-2 (Bio-Rad) were used to remove residual Triton X-114 from the protein-containing layer following the manufacturer’s protocol. The Triton X-114 treated lipases were then incubated with poly(ε-lysine) resin for further endotoxin removal following the manufacturer’s protocol (Pierce).

### Purification of SitC-6xHis

SitC-6xHis was purified as previously described with a few modifications [50]. Plasmid pOS1-*P*_*sarA*_*-sod*_*RBS*_*-sitC-6xhis* was transformed into a Δ*bmfBB* mutant strain to generate Δ*bmfBB* + pOS1-*P*_*sarA*_*-sod*_*RBS*_*-sitC-6xhis*. Two-liter cultures of Δ*bmfBB* + pOS1-*P*_*sarA*_*-sod*_*RBS*_*-sitC-6xhis* were grown in medium supplemented with 2MB (9 mM), egg yolk LDL (0.1% w/v), or GTO (50 μM) prior to purification. All other steps of the protocol remained the same.

### Geh-6xHis and Sal1-6xHis lipoprotein processing

20 μM purified SitC was incubated with 1 μM endotoxin-free Geh-6xHis or Sal1-6xHis at 37°C for two hours. Reactions were stopped by freezing at −80°C. Geh- or Sal1-mediated effects on lipoprotein-induced immunostimulatory activity were assayed upon addition of SitC to macrophages and quantifying cytokine production after 16 hours using a customized cytometric bead array flex kit (BD Biosciences) and flow cytometry (LSR Fortessa cell analyzer) as previously described [43,50].

### Egg-yolk LDL extraction

Egg-yolk LDL was extracted as previously described with the following modifications [19]. Briefly, after careful removal of the vitellin membrane, the yolk was collected and mixed with 1X PBS (vol/vol, 1:2) for one hour with stirring at 4°C. Plasma was collected by centrifugation at 10,000 × g for 45 minutes at 4°C and livetins were removed from the plasma by precipitation with 40% ammonium sulfate at pH 8.7 for one hour at 4°C followed by centrifugation at 10,000 × g at 4°C for 45 minutes. The supernatant was collected followed by removal of ammonium sulfate through dialysis against distilled water overnight. LDL was then collected by centrifugation at 10,000 × g for 45 minutes at 4°C.

### Cell-free supernatant collection

Bacterial strains were grown in 3 mL fresh RPMI overnight at 37°C. Bacteria were sub-cultured (1:100) in 3 mL fresh RPMI medium +/- 50 μM GTO (Sigma-Aldrich) or 0.34 μg/μL purified human LDL (Kalen Biomedical) and grown for eight hours at 37°C. The OD_550_ was measured prior to pelleting bacteria by centrifugation at 3000 × g for 15 min. Cell free supernatant was collected after filtration through a 0.22-μm pore size PES membrane filter (Corning) and subsequently stored at −80°C.

### Macrophage culture and *in vitro* macrophage activation assay

Murine BMM were derived from bone marrow progenitor cells that were isolated from tibias and femurs of WT C57BL/6 mice (JAX stock no. 000664), or TLR2^-/-^ mice (JAX stock no. 004650) as previously described [43,50]. 65,000 BMM were seeded into a 96-well plate in 90 μL BMM medium supplemented with streptomycin (100 μg/mL) and gentamycin (10 μg/mL), followed by incubation for 24 hours at 37°C, 5% CO_2_ prior to addition of SitC or bacterial cell-free supernatant. SitC (at the indicated concentrations) +/- pretreatment with endotoxin-free Geh or Sal1 or OD_550_ normalized *S*. *aureus* cell-free supernatant (10 μL), with equivalent protein content as determined by SDS-PAGE, was applied to macrophages. After 16 hours, macrophage supernatant was collected and the secreted cytokine and chemokine profiles were measured using a customized cytometric bead array flex set (BD Biosciences) and flow cytometry (LSR Fortessa cell analyzer) as previously described [43,50]. Each macrophage activation assay was conducted three times in triplicate using macrophages derived from at least two different mice and two separate preparations of SitC to assess reproducibility. As a result, the magnitude of maximal cytokine produced varies between separate experiments and figure panels. All cytokine measurements within a single graph are from the same experiment.

### pNp-15:0, pNp-18:1, and pNp-4:0 hydrolase activity assays

The lipase activity of Geh and Sal1 was assessed in 96-well flat bottom plates as previously described with some modifications [72]. The pNp esters, pNp-15:0 and pNp-18:1, were synthesized by Avanti Polar lipids whereas pNp-4:0 was purchased from Sigma. Stock solutions (16 mM) of the substrates were prepared in isopropanol followed by dilution (1:10) into reaction buffer (50 mM Tris·HCl, 1 mg/mL arabic gum, 0.05% Triton X-100, pH 8.0). Seven 10-fold serial dilutions were made in reaction buffer containing 10% isopropanol and hydrolysis of the substrates was initiated by adding Geh (2 nM) or Sal1 (2 nM) in a final volume of 100 μL. Color change was measured at OD_410_ every 10 seconds for 15 min at 25°C in the dark using a BioTek Synergy H1 plate reader alongside a para-nitrophenol (Sigma) standard curve. The assay was performed in triplicate and reaction rates were calculated from composite data derived from 6–7 independent assays. Reaction rates were plotted against the corresponding substrate concentrations and fitted to a standard Michaelis-Menten curve for determination of *K*_m_ using GraphPad Prism 9.

### Murine systemic infection model

Overnight cultures of WT, Δ*bmfBB*, Δ*fakB2*, Δ*bmfBB* Δ*fakB2*, and Δ*bmfBB* Δ*fakB2* + *fakB2* strains were subcultured (1:100) in 15 mL TSB supplemented with BCFA precursors (10 mM IBA, 9 mM IVA, and 9 mM 2MB) for 3 hours at 37°C with shaking. After centrifugation at 3,234 × g for 15 minutes, bacterial pellets were washed three times in 5 mL of 1X phosphate buffered saline (PBS) and adjusted to an OD_600_ value of 0.32 (∼1 × 10^8^ cfu/mL). The infection dose was confirmed by plating serial dilutions of the inoculum on tryptic soy agar. 5-6-weeks old C57BL/6J mice (JAX stock no. 000664) or B6.129-*Tlr2*^*tm1Kir*^/J (*TLR2*^*−/−*^; JAX stock no. 004650) mice were infected by retro-orbital injection with 1 x 10^7^ CFU in 100 μL. At 24 hours or 72 hours post infection, hearts, kidneys, livers, and spleens were collected and homogenized in 5 mL of PBS and serial dilutions of organ homogenates were plated on tryptic soy agar to quantify bacterial CFU. When serial dilutions had no detectable bacteria, organ CFU was enumerated from direct plating of homogenates on tryptic soy agar.

### Bacteria total lipid extraction

Total lipids were extracted from *S*. *aureus* using the method of Bligh and Dyer [73]. Briefly, overnight cultures were diluted 1:100 into 50 mL RPMI, with or without GTO or BCFA precursor supplementation and grown for 8 hours at 37°C with shaking. Bacteria were washed three times with 0.9% NaCl, followed by centrifugation at 5,000 × g for 15 minutes at 4°C and storage at −80°C. For lipid extraction, the pellets were mixed with 10 mL methanol (Fisher)-chloroform (Alfa Aesar) solution (2:1, vol/vol) and incubated at room temperature on a shaker for 2 hours. The lipid-containing layer was isolated after centrifugation at 5,000 × g for 15 minutes at 4°C. The extraction was repeated twice using 10 mL methanol-chloroform-water solution (2:1:0.8, vol/vol/vol). 7 mL of chloroform and 7 mL of water were added in sequential order to the lipid solution in a separatory funnel and separation was allowed to occur overnight. The lipid-containing layer was collected the next day and dried under nitrogen gas.

### Fatty acid extraction and GC-FAME analysis

Dried lipids (prepared as described above) were sent to Avanti Polar lipids for fatty acid extraction and GC-FAME analysis. Briefly, lipid samples were hydrolyzed and methylated in 25% (weight) sodium methoxide to produce fatty acid methyl esters (FAME). 50 μL of each sample was then assayed by gas chromatography on an Agilent Technologies 7890A gas chromatograph with FID autosampler. FAMEs detected in samples were identified based on their column retention times as compared to a set of standards containing C4 to C24:1 FAMEs.

### Statistical analysis

All experiments were repeated at least three independent times. For *in vitro* macrophage data, statistical significance was analyzed from representative experiments conducted in triplicate. Each triplicate experiment was repeated a minimum of three independent times. All statistical significance was analyzed using Prism version 9.0 with statistical tests specified in the figure legends. Statistical analyses on data derived from animal studies, used non-parametric Kruskal- Wallis (Fig 6) or Kolmogorov Smirnov (Fig 7) tests. The Kolmogorov Smirnov test was chosen to analyze data in Fig 7 due to its sensitivity to changes in the cumulative distribution between groups. The number of animals per treatment group is indicated as “n” in the figure legends. The group size for a Δ*bmfBB* mutant was increased on account of the highly variable infection patterns for this strain. For all other cytokine data, statistical significance (*P* < 0.05) was determined by one-way ANOVA with Tukey’s post-hoc test.

## Supporting information

S1 Text Table A Strains used in this study. Table B List of oligonucleotides used in this study.
(DOCX)
