# Supplementary material for: Branched chain fatty acid synthesis drives tissue-specific innate immune response and infection dynamics of Staphylococcus aureus
Source: PLoS Pathog. 2021 Sep 8;17(9):e1009930. doi: 10.1371/journal.ppat.1009930 (PMC8452012; doi:10.1371/journal.ppat.1009930)
Supplement: S1 Text — Table A Strains used in this study. Table B List of oligonucleotides used in this study. (DOCX) [file ppat.1009930.s001.docx]

Table A. Strains used in this study.

|  | Genotype | Designation | Reference |
| --- | --- | --- | --- |
| USA300 AH-LAC | WT USA300 (AH-1263) parent strain, plasmid cured. | AH-LAC (WT) | [1] |
| FA-S973 | AH-LAC with an in-frame gene replacement Δ*geh::kan* | Δ*geh* | [2] |
| FA-S987 | AH-LAC Δ*geh* transduced with pJC1112-*geh* | Δ*geh+geh* | [2] |
| FA-S1399 | AH-LAC with an in-frame deletion of *sal1* | Δ*sal1* | [2] |
| FA-S1488 | AH-LAC Δ*sal1* transduced with pJC1111-*sal1* | Δ*sal1*+*sal1* | [2] |
| FA-S1490 | AH-LAC Δ*sal1* transduced with Δ*geh::kan* | Δ*geh* Δ*sal1* | [2] |
| FA-S2232 | AH-LAC Δ*fakB2* | Δ*fakB2* | [3] |
| FA-S2230 | AH-LAC Δ*fakB1* | Δ*fakB1* | [3] |
| FA- S2232 | AH-LAC Δ*fakB2* transduced with pJC1111-*fakB2* | Δ*fakB2*+*fakB2* | This study |
| FA- S2230 | AH-LAC Δ*fakB1* transduced with pJC1111-*fakB1* | Δ*fakB1*+*fakB1* | This study |
| FA-S2192 | AH-LAC with an in-frame gene replacement Δ*bmfBB*::*kan* | Δ*bmfBB* | [3] |
| FA-S2244 | AH-LAC Δ*bmfBB::kan* with an in-frame deletion of *fakB2* | Δ*fakB2* Δ*bmfBB* | [3] |
| FA-S2260 | AH-LAC Δ*bmfBB::kan* Δ*fakB2* transduced with pJC1111-*fakB2* | Δ*fakB2* Δ*bmfBB + fakB2* | [3] |
| FA-S2094 | AH-LAC Δ*bmfBB*::*kan* containing pOS1- *P_sarA_-sod_RBS_*-*sitC-6xHis* | Δ*bmfBB P_sarA_-sod_RBS_*-*sitC-6xHis* | This study |
| FA-S1324 | T7 Express *E. coli* *lysY/I^q^* containing pQE60-*geh-6xHis* | *lysY/I^q^* pQE60-*geh-6xHis* | [2] |
| FA-S1551 | AH-LAC transduced with NE1757 transposon containing erm resistance and disrupting *lspA* | *lspA::tn* | [4] |
| RN4220 | Restriction deficient *S. aureus* strain for plasmid passage | RN4220 | [5] |
| RN9011 | RN4220 with SaPI-1 integrase expressing pRN7203 plasmid | RN9011 | [6] |
| DH5α | *E. coli* strain for passaging recombinant pIMAY and pJC plasmids | DH5α | NEB, Cat # C2989K |
| DC10B | *E. coli* strain for passaging recombinant pIMAY and pJC plasmids | DC10B Δ*dcm* | [7] |
| T7 Express *lysY/I^q^* | *E. coli* strain for expressing recombinant Geh-6xHis and Sal1-6xHis | *lysY/I^q^* | NEB, Cat# C3013I |

Table B. List of oligonucleotides used in this study.

| **Name** | **Sequence** |
| --- | --- |
| fakB1SOE1-KpnI | ATAT- GGTACC(KpnI)-AGCACCGCTATAGGCG |
| fakB1Comp SOE2 | GGTCATCACAGCAATTTTCATGGGTTTCACTCTCCTTCTAC |
| fakB1Comp SOE3 | GTAGAAGGAGAGTGAAACCCATGAAAATTGCTGTGATGACC |
| fakB1Comp SOE4-SacI | ATAT-GAGCTC(SacI)-AACCTCATTTCAACGTAAAAAAGAG |
| fakB2 SOE1-KpnI | ATAT-GGTACC(KpnI)-ATAATTATGACTTTATCCATTCTAGTTGCACATG |
| fakB2 Comp SOE2 | GAGTCTGTTACTATAATCTGTTTTGTCATGGGTTTCACTCTCCTTCTAC |
| fakB2 Comp SOE3 | GTAGAAGGAGAGTGAAACCCATGACAAAACAGATTATAGTAACAGACTC |
| fakB2 Comp SOE4-SacI | ATAT-GAGCTC(SacI)-GATGTAAATGGTTTAACCATGCAC |
| pQE60-Sal1 NcoI | ATAT-CCATGG(NcoI)-CCAAAGCGAAAGATGATCAAAC |
| pQE60-Sal1 BglII | ATAT-AGATCT(BglII)-TGCTTGCTTAGTATCAGTC |

References

1. Boles BR, Thoendel M, Roth AJ, Horswill AR. Identification of Genes Involved in Polysaccharide-Independent Staphylococcus aureus Biofilm Formation. Ratner AJ, editor. PLoS ONE. 2010 Apr 14;5(4):e10146.

2. Chen X, Alonzo F. Bacterial lipolysis of immune-activating ligands promotes evasion of innate defenses. Proc Natl Acad Sci USA. 2019 Feb 26;116(9):3764–73.

3. Teoh, WP, Chen, X, Laczkovich, I, and Alonzo, F. (2021). *Staphylococcus aureus* adapts to the host nutritional landscape to overcome tissue-specific branched-chain fatty acid requirement. Proc. Natl. Acad. Sci. *118*, e2022720118.

4. Grayczyk JP, Harvey CJ, Laczkovich I, Alonzo F. A Lipoylated Metabolic Protein Released by Staphylococcus aureus Suppresses Macrophage Activation. Cell Host & Microbe. 2017 Nov;22(5):678-687.e9.

5. Fairweather N, Kennedy S, Foster TJ, Kehoe M, Dougan G. Expression of a cloned Staphylococcus aureus alpha-hemolysin determinant in Bacillus subtilis and Staphylococcus aureus. Infect Immun. 1983 Sep;41(3):1112–7.

6. Chen J, Yoong P, Ram G, Torres VJ, Novick RP. Single-copy vectors for integration at the SaPI1 attachment site for Staphylococcus aureus. Plasmid. 2014 Nov;76:1–7.

7. Monk IR, Shah IM, Xu M, Tan M-W, Foster TJ. Transforming the Untransformable: Application of Direct Transformation To Manipulate Genetically Staphylococcus aureus and Staphylococcus epidermidis. Novick RP, editor. mBio. 2012 Mar 20;3(2):e00277-11.
